# Establishment of a new representative model of human ovarian cancer in mice

**DOI:** 10.1186/1757-2215-6-9

**Published:** 2013-02-06

**Authors:** Jianjun Zhang, Xinlian Chen, Gang Shi, Xiaoyan Xie, Hongqian Liu, Xuemei Zhang, Yi Lai, Yan Zuo, Zhong Chen, Shanling Liu, He Wang

**Affiliations:** 1 Laboratory of Genetics, West China Institute of Maternal and Child Health, West China Second University Hospital, Sichuan University, Chengdu 610041, P. R. China; 2 Laboratory of Cell and Gene Therapy, West China Institute of Maternal and Child Health, West China Second University Hospital, Sichuan University, Chengdu 610041, P. R. China; 3 Department of Obstetrics and Gynecology, West China Second University Hospital, Sichuan University, Chengdu, 610041, P. R. China; 4 Prenatal Diagnosis Center of Sichuan Province, West China Second University Hospital, Sichuan University, Chengdu, 610041, P. R. China; 5 Key Laboratory of Obstetrics, Gynecology, Pediatric Diseases and Birth Defects of Ministry of Education, Chengdu, 610041, P. R. China; 6 Division of Medical Genetics, University of Utah School of Medicine, Salt Lake City, Utah 84132, USA

**Keywords:** Human, Ovary carcinoma, Animal model, CA125, Intraperitoneal

## Abstract

**Background:**

Intraperitoneal (i.p.) models that accurately mimic the feature behavior of human ovarian cancer are required to investigate the pathology and therapeutics of the disease. However, established i.p. models which are well-characterized and reliable are few. The purposes of this study are to establish a representative mice i.p. model of the disease and to analyze the consequent pathology.

**Methods:**

Fresh tumor cells fiom the ascites of patient were injected into female NOD/SCID mice intraperitoneally. Histology, Cytogenetic, immunohistochemistry,tumor markers of CA125,AFP, CA-199 and CEA were used to analyze the model.

**Results:**

The mice developed marked abdominal distention within 6 months after inoculated with tumor cells from a patient with epithelial ovarian carcinoma. The mice developed clinically evident intraperitoneal tumors and massive ascites containing numerous tumor cells in clumps. CA125 level in our model was high in both serum and ascites supernatants, while levels of other tumor markers, such as AFP, CA-199 and CEA, were normal. Cytogenetic analysis and immunohistochemical staining confirmed its characteristics resembling human epithelial ovarian tumor.

**Conclusions:**

The model described in this paper accurately mimics the features of ovarian tumor, which may be useful for evaluation of new therapeutics.

## Background

Ovarian cancer is the leading cause of death among gynecologic tumors with a death toll up to 13850 in the USA by 2010 [1]. Epithelial ovarian cancer (EOC) accounts for over 90% of all ovarian malignancies. Its high mortality is attributable to the fact that 75% of the patients are not diagnosed until the advanced stage. Although the majority of the patients respond to initial chemotherapy after a primary debulking surgery, most eventually experience recurrence as they become chemoresistant [2]. Research into the development of well-characterized and reliable models is crucial for evaluating efficacy of novel therapeutics, which may help improve patient survival.

In developing an *in vivo* model of human ovarian neoplasia, it is critical to ensure that the model mimics the behavior of ovarian tumor in patient accurately. Research teams have attempted relevant models employing subcutaneous (s.c.) and intraperitoneal (i.p.) xenografts in immunodeficient mice [3-16], where only the i.p. models were in line with the clinical manifestations in the advanced stage given the carcinomatosis in the peritoneal cavity with large volumes of ascites. Further more, such models seemed clinically useful in demonstrating efficacy of the intraperitoneal therapies being tested, which was hardly the case with the s.c. models. Most of the models commonly used in ovarian cancer research are based on established cell lines. However, it is found that, compared with the cell lines, only xenografts established directly from fresh human ovarian tumor tissues could match the original tumors in terms of antigen gene expression [12], as may be attributed to the fact that the cell lines could have changed their protein expression patterns and lost the heterogenetic characteristics of human cancer through long-term *in vitro* culturing.

Serum tumor markers also seem useful in the management of several types of ovarian tumor. For example, CA125, measurable in the serum, is routinely used as a diagnostic biomarker of ovarian cancer in clinical settings, which, however, due to its low sensitivity in the early stage, tends to make better sense for monitoring tumor progression and response to therapy. Its use for evaluating therapeutic efficacy has been attempted in a few studies [17,18].

In our study, we employed the NOD/SCID mice and fresh tumor cells, and established a novel reproducible xenograft model of ovarian cancer, which is transplantable and is characterized by its close mimic of the progressive massive ascites, extensive intra-abdominal carcinomatosis, and elevated CA125 levels in both blood and ascites.

## Methods

### Clinical characteristics

The patient was a 53-year-old post-menopausal woman presenting with a 2-month history of abdominal distention with a mass in the right adnexa. Cytological study of the ascites indicated presence of adenocarcinoma cells, with a serum CA125 level of 1364 U. She received intraperitoneal chemotherapy with Thiotepa twice (1^st^: 30mg, 2^nd^: 20mg) before surgery (cyto-reductive surgery, extrafascial hysterectomy, bilateral adnexectomy, omentectomy, appendectomy). Poorly differentiated serous adenocarcinomas of the ovary (stage FIGO IIIC) were pathologically confirmed. Informed consent was acquired from the patient.

### Animals

Four- to six-week-old female NOD/SCID mice (purchased from Beijing HFK Bio-Technology Co. Ltd., Beijing, China) were housed in sterile micro-isolators (5 mice per cage). Feeding and water were given *ad libitum*. All procedures were performed under sterile conditions in a laminar flow hood. The animals were monitored daily for general health status. All animal experiments described in this study were approved by the Institutional Animal Care and Use Committee of Sichuan University.

### Heterotransplantation and in vivo Passaging

A tumor specimen (ascites) was collected and sterilely transported on ice to the laboratory. The ascites was centrifuged at 300G for 5min. The cell pellet was immediately intraperitoneally injected into the NOD/SCID mice. The number of tumor cells from the patient inoculated into the two mice was 5x10E7.

When the animals had marked abdominal distensions, their ascites was collected from the tumor-bearing mice, and directly injected into the next mice. The mice were then monitored daily for general health status as well as the degree of abdominal extension before another passaging. Xenografts were established within 6 months after heterotransplantation in both mice.As passaging continued, the number of tumor cells we used was 1x10E7. The peritoneal cavity of each animal was examined. Tissues and organs suspected of being affected by tumor were eventually harvested for histological examinations.

### Freezing storage of xenograft tumor

After the animals were sacrificed, ascites was collected and centrifuged at 300G for 5min. The pellet was suspended in cold freezing media, consisting of DMEM with 8% DMSO and 20% fetal bovine serum (FBS). The cells were then transferred to liquid nitrogen for long-term storage. For retransplantation, the tumor suspension underwent a rapid thaw at 37°C, and was diluted in 10ml of DMEM without FBS. Then it was centrifuged, and intraperitoneally injected into the mice.

### Immunohistochemistry

Tumors in the peritoneal cavity were fixed in 4% neutral buffered formalin, and processed for histological examination. The fixed samples were embedded in paraffin, 5μ-thick sections were cut, and H-E staining was applied. Cell suspensions from ascites were cyto-centrifuged, and smear was fixed with cold acetone. Endogenous peroxidase activity was then blocked with 3% hydrogen peroxide in PBS for 10min. The samples were subsequently rinsed with PBS for 5min. All tissues were blocked with BSA for 20min at room temperature. Primary monoclonal antibodies to vimentin (dilution 1:300), pan-cytokeatin (dilution 1:400), EMA (dilution 1:500), P53 (dilution 1:300), MMP-2 (dilution 1:300) (all supplied by Wuhan Boster Bio-Engineering Co. Ltd., Wuhan, China), and CA125 (dilution 1:400) (supplied by Beijing Zhongshan Golden Bridge Biotechnology Co. Ltd., Beijing, China) were used. Sections were incubated with the antibodies overnight at 4°C following three 5-min washes with PBS, then incubated with biotinylated secondary antibody at 37°C for 20min, and finally incubated with streptavidin-biotin-horse radish peroxidase complex at 37°C for 20min. The sections were then developed with diaminobenzidine (DAB) as a substrate. Cellular nuclei were counterstained with hematoxylin. All operations followed the manufacturers’ recommendations. Absent primary antibody during processing was used as blank control. Immunostaining was semi-quantitative as described [19].

### Analysis of tumor marker in blood and Ascites

Whole blood and ascites specimens were collected from the tumor-bearing mice, and centrifuged for 10min. The supernatants were isolated and stored at −20°C for subsequent analysis. Levels of CA125, CA199, AFP and CEA were determined.

### Cytogenetics

Ascites specimens were collected and centrifuged. Cell pellet was resuspended in DMEM supplemented with 10% heat-inactivated fetal calf serum (FCS), and exposed to 0.5μg/ml demecolcine (Colcemid) for 2 hours. Cytogenetic analysis was accomplished by G-banding using a conventional Giemsa staining protocol [20]. Briefly, tumor cells were harvested and incubated in 75mM potassium chloride at 37°C. Then they were fixed with 25% acetic acid in anhydrous methanol twice. The slides were trypsinized and stained with 10% Giemsa, and 35 metaphase spreads were karyotyped.

## Results

### Growth of human ovarian tumors in scid mice

**Figure 1:**
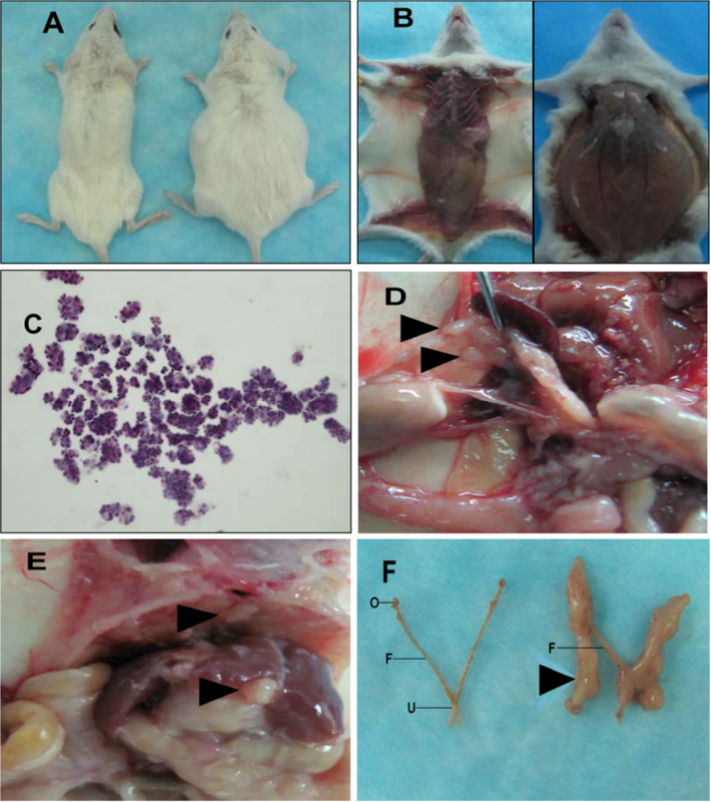
**(A) Female NOD/SCID mice with abdominal distention after injection of ascites tumor cells. **Left image is non-injected control; (**B**) Presence of milky ascites in mice; (**C**) Large, crowded clumps of cells in ascites; (**D**) Mesentery invaded by tumor tubercles; (**E**) Sub-diaphragmatic tumor deposits and deposits on liver surface; (**F**) Reproductive system affected by tumor. Left image is non-injected control. Arrowhead indicates metastatic tumor. O-ovary; S-fallopian tube; U-uterus.

Tumor cells in the ascites from the patient with poorly differentiated ovary papillary serous adenocarcinoma were injected into the peritoneal cavities of female NOD/SCID mice. Xenografts were established within 6 months after heterotransplantation. As passaging continued, the time to achieve a clinically evident disease decreased to 1–2 months. All mice eventually developed visible abdominal swelling (Figure 1A) and yellowish pale ascites (Figure 1B). The volume of ascites was generally 4 to 8ml per mouse. Abundant cells of clinical resemblance were seen individually or in clusters on cytological examination of the ascites (Figure 1C). The malignant features of the cells were confirmed by high nuclear-cytoplasmic ratio, nuclear multiforme and predominant nucleoli; and these histological characteristics were documented in different mouse generations. The mean viable tumor cell yield from ascites was approximately 1.5 × 10^9^ cells per mouse. The malignant cells in the ascites could be reproducibly maintained and directly passaged to subsequent hosts. The cells have been serially transplanted 8 times to date. This model may be reproduced using our cryopreserved tumor cells in liquid nitrogen free of histological changes.

### Metastatic pattern

The metastatic pattern was investigated, where similar results were noted in different generations. In the advanced stage of this model, the mesentery was most frequently invaded by tumor tubercles (Figure 1D). Deposits of tumor cells were found on the surfaces of peritoneal organs, such as the sub-diaphragmatic and liver linings, which replicated the initial mice (Figure 1E). The reproductive system was severely affected by tumor cells (Figure 1F). Severe unilateral or bilateral ovarian pathogenesis was found in 75% of the animals in our model. In most cases, the histomorphology and cytology of ovary changed as the condition developed from crowding and displacement to complete invasion. All suspicious areas were biopsied. No evidence of extraperitoneal spread was noted.

### Cytogenetics

**Figure 2:**
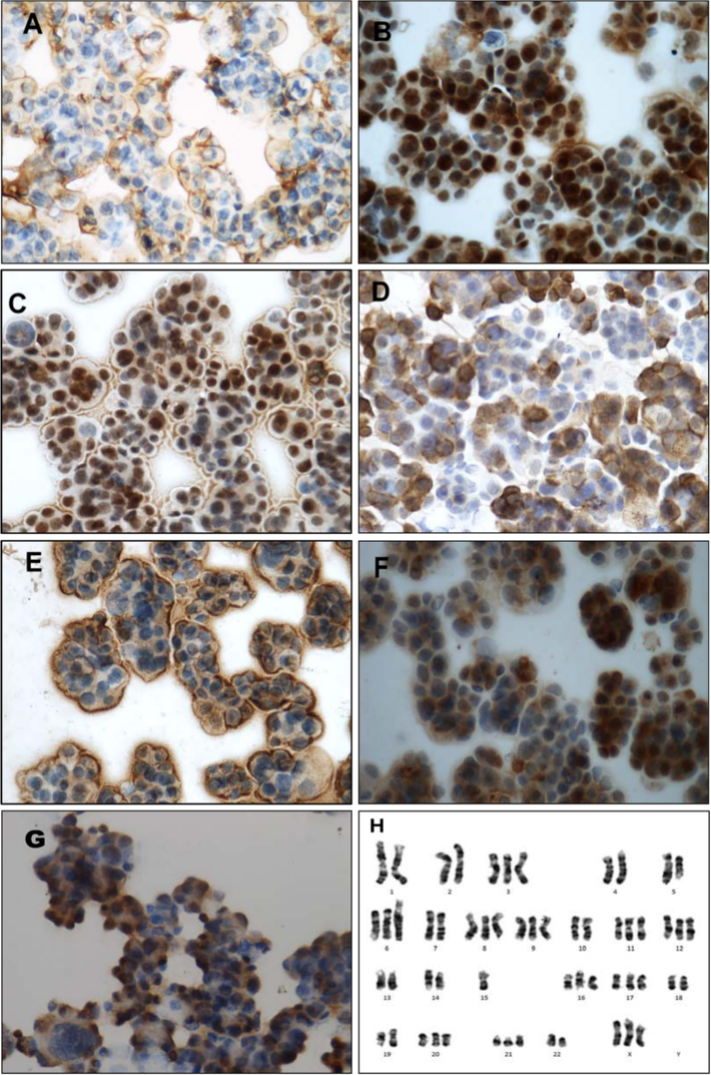
**Immunohistochemical staining and Karyotyping profile of xenograft tumors from ascites of the sixth generation. **(**A**) EMA; (**B**) PCNA; (**C**) P53; (**D**) Vimentin; (**E**) CA125; (**F**) Pan-cytokeatin; (**G**) MMP-2 (x400); (**H**) Karyotyping profile of malignant cells from ascites. Number of chromosomes generally between 54 and 60, mostly triploidy with structural abnormalities.

Human female origin of the established xenograft model was confirmed by cytogenetic analysis. Thirty-five banded metaphases were analyzed. Chromosome number ranged from 54 to 60 (median: 58), mainly trisomy (Figure 2H).

### Immunohistochemistry

To analyze the histopathology of the tumor cells in the ascites, immunohistochemical staining was performed to determine expression of epithelial membrane antigen (EMA), Pan-cytokeatin, vimentin (VIM), PCNA, P53, CA125 and MMP-2. The results are given in Table 1 and Figure 2. It’s found that there is no significant changes in comparison with the first generation (the pictures were showed as Additional file 1: Figure S3).

**Table 1:** Immunohistochemical markers of heterotransplanted tumor cells from ascites of the sixth generation

**Marker**	**Result**	**Method**
CA125	+ +	IHC *
EMA	++	IHC
Pan-cytokeratin	++	IHC
VIM	++	IHC
PCNA	++++	IHC
P53	+++	IHC
MMP-2	++	IHC

* IHC: Immunohistochemical staining.

### Tumor makers in serum and ascites

In analyzing the CA125, CA199, CEA and AFP levels in serum and ascites, we found that only the CA125 level rose in both serum and ascites in the tumor-laden mice. The serous CA125 level ranged 293.4-1024.4U/ml. Supernatant of the malignant ascites was found to contain 484.2-3800U of CA125 per ml. The normal serous CA125 level (< 11 U/ml) was documented in all healthy control mice.

## Discussion

We established a reproducible murine xenograft model of ovarian carcinoma employing tumor cells harvested from the ascites of an ovarian cancer patient, where the cells may be reproducibly maintained and directly passaged to subsequent hosts. This model requires no selective methods before intraperitoneal growth such as enzymatic digestion, selective anchorage-independent growth, and subcutaneous inoculation [7,12,15,16]. As a result, the unwanted implications of the manipulations were avoided.

The tumor cells from the ascites were hyperchromatic for CA125 antigen, Pan-cytokcratin and epithelial antigen, which confirmed it to be epithelial differentiation [8]. MMP-2 is believed to be involved in two aspects of the ovarian cancer spread process: It helps tumor cells penetrate the basement membrane of the ovary to invade the stroma [21], and may enable the tumor cells to detach from the epithelial surface and migrate into the peritoneal cavity [22,23]. It is reported that the rate of MMP2 expression in ovarian cancer is high and irrelevant with either clinical staging or histological typing [24]. One possible explanation was the grave malignance of ovarian cancer. In our model, we also noted tumor cells with high MMP-2 expression. P53 mutation occurs early in the progression of ovarian cancer [25], which is found in some 50% or more of advanced serous adenocarcinomas while rarely noted in earlier stages [26-28]. In this model, tumor cells show intense expression of p53, consistent with the previous reports.

We examined the transplanted tumors in ascites at different passages, and found that the characteristics did not change from one generation to another. As a result, this may offer an unlimited *in vivo* supply of stable tumor cells, which may be of great value for ensuring reproducibility and reliability of research results. In the future, we may establish similar models of other ovarian cancer subtypes for furthering our knowledge on the disease.

In this model, we examined the CA125, CA199, CEA and AFP levels in blood and ascites, only to find that the CA125 level alone rose in the tumor-bearing mice. This makes a possibility where CA125 may be used as a biomarker in the model, similar to its clinical application, for prognosis prediction and therapeutic evaluation in future studies.

## Conclusions

This novel model of ovarian cancer, which mimics human carcinosis accurately, appears simple and reproducible. It may also be instrumental for culturing ovarian carcinomas *in vivo*, hence as an unlimited source of ovarian cancer cells for research purposes. Great merit is seen in our model for evaluating new therapeutics given its CA125 level matching the human situation closely.

## Competing interests

We have no proprietary, financial, professional or other personal interest of any kind in any product, service and/or company that could be construed as influencing the position presented in the manuscript entitled:Establishment of a new representative model of human ovarian cancer in mice.

## Authors’ contributions

JJ Z carried out the heterotransplantation and in vivo passaging of the model, participated in in its design and drafted the manuscript. HQ L, XM Z, XY X carried out the immunoassays. XL C, YL participated in the Cytogenetics study. GS, YZ participated in the collection and transport of clinical specimens and heterotransplantation. ZC ,SL L, HW conceived of the study, and participated in its design and coordination and helped to draft the manuscript. All authors read and approved the final manuscript.

## Supplementary Material

Additional file 1 **Immunohistochemical staining a of xenograft tumors from ascites of the first generation. **(A1) EMA; (B1) PCNA; (C1) P53; (D1) Vimentin; (E1) CA125; (F1) Pan-cytokeatin; (G1) MMP-2 (x400). Click here for file
