# Supplementary material for: Establishment of a new representative model of human ovarian cancer in mice
Source: J Ovarian Res. 2013 Feb 6;6:9. doi: 10.1186/1757-2215-6-9 (PMC3573975; doi:10.1186/1757-2215-6-9)
Supplement: Additional file 1 — Immunohistochemical staining a of xenograft tumors from ascites of the first generation. (A1) EMA; (B1) PCNA; (C1) P53; (D1) Vimentin; (E1) CA125; (F1) Pan-cytokeatin; (G1) MMP-2 (x400). [file 1757-2215-6-9-S1.pdf]

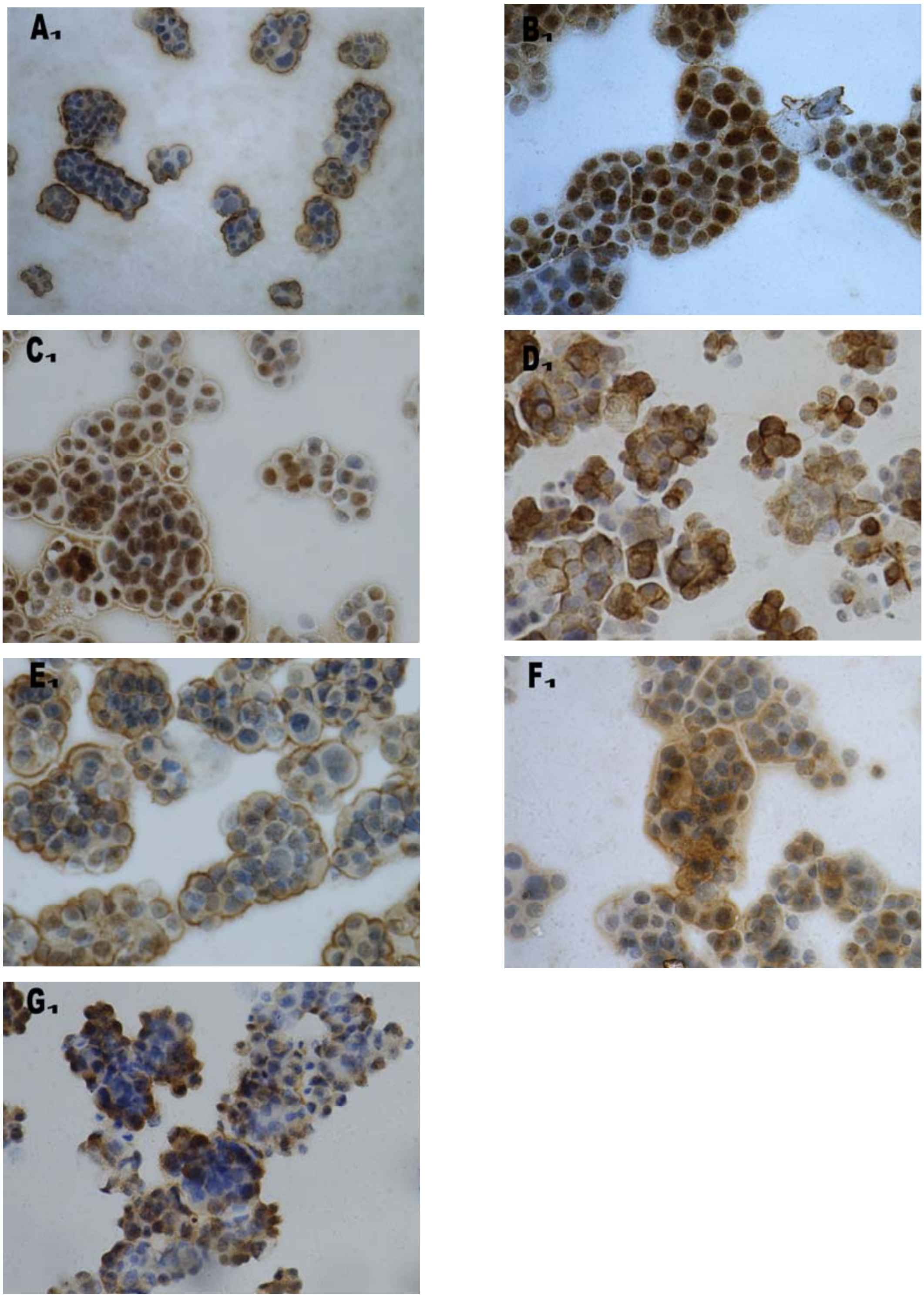

Fig 3

Immunohistochemical staining a of xenograft tumors from ascites of the first generation. (A1) EMA; (B1) PCNA; (C1) P53; (D1) Vimentin; (E1) CA125; (F1) Pan-cytokeatin; (G1) MMP-2 (x400);
